# How to sanction international wrongdoing? The design of EU restrictive measures

**DOI:** 10.1007/s11558-022-09458-0

**Published:** 2022-02-24

**Authors:** Katharina Meissner

**Affiliations:** Department of Political Science, Centre for European Integration Research (EIF), University of Vienna, Vienna, Austria

**Keywords:** European Union, Foreign policy, Sanction, Salience, Threat

## Abstract

Sanctions are among the most widely used foreign policy tools of governments and international organizations in response to national or international wrongdoings. Beyond the dichotomous question of whether to adopt or not to adopt sanctions against a target, decision-makers develop different designs when they impose restrictions: targeted sanctions like asset freezes and travel bans, arms embargoes, or economic sanctions such as financial restrictions and commodity bans. What accounts for this variation in the design of sanctions regimes? This article investigates this question by developing a configurational explanation that combines domestic- and international-level factors for the choice of an economic versus a targeted sanctions design. I test these factors on original data mapping European Union (EU) autonomous sanctions against third countries in force in 2019 through set-theoretic methods. The analysis shows that a militarily strong target’s serious misbehavior through grave human rights violations triggers EU action in the form of economic sanctions, however, only in combination with two conditions: first, the EU reacts to a misbehavior through the adoption of an economic design when the United States imposes economic sanctions, too (path 1); second, the salience of a target’s conflict triggers an economic design of sanctions in case of grave human rights violations (path 2).

**Supplementary Information:**

The online version contains supplementary material available at 10.1007/s11558-022-09458-0.

## Introduction

Scholars have been studying the effectiveness of sanctions by states and international organizations for decades (e.g. Baldwin & Pape, 1998; Biersteker et al., 2013; Dashti-Gibson et al., 1997; Hufbauer et al., 2007; Giumelli, 2011; Portela, 2010). Still, no clear consensus has yet emerged under what conditions sanctions are successful. One condition for successful sanctions appears to be their design (Brooks, 2002; McLean & Whang, 2014: 590).

Yet, the design of sanctions has received far too little scholarly attention (exceptions are Giumelli, 2011; Hedberg, 2018; McLean & Whang, 2014; see also Bapat et al., 2020: 449). Arguably, states and international organizations have a wide variety of sanctioning instruments at their disposal: arms embargoes; asset freezes; broad commercial, dual-use and financial restrictions; travel bans; or interruption of services. One of the most discussed design choices in the literature is the variation between so-called smart or targeted sanctions versus economic restrictions such as commodity embargoes or financial restrictions (e.g. Brooks, 2002; Drezner, 2011). Nevertheless, empirical research hitherto primarily examines whether and why a sender adopts or does not adopt sanctions (Hafner-Burton & Montgomery, 2008; von Soest & Wahman, 2015). By relying on such a dichotomous variable, scholars overlook the broad variation senders have when they design sanctions (see also Hedberg, 2018). This article fills this void in the literature by systematically investigating the design of sanctions on a new, author-created dataset mapping European Union (EU) autonomous sanctions against third countries in force in the year 2019. As the empirical data in this article shows, the EU’s recent more frequent use of economic sanctions is not unique to Europe or the current time period. Rather, we observe similar economic sanctions, based on financial restrictions and commodity bans, in earlier sanctions regimes of the EU (Giumelli et al., 2020) and in programs designed by other actors like the United Nations (UN) (Biersteker et al., 2018; Targeted Sanctions Dataset (TSC)) and the United States (US) (Weber & Schneider, 2020).

Understanding the design choices when organizations such as the EU adopt sanctions is anything but trivial since such decisions bear consequences for domestic and international actors. On the one hand, recent research identifies the design of sanctions as key to their ultimate success in provoking a change of the target’s behavior (McLean & Whang, 2014). Increasing the costs for the target through an appropriate design of restrictive measures is shown to have a positive effect on sanctions’ effectiveness (Bapat et al., 2020: 451). On the other hand, especially an economic design of sanctions can bear unintended consequences both for the target as well as the for the sender state(s). Scholarship documents the negative externalities of economic sanctions regarding human rights, democracy, and the rule of law in target countries (Allen et al., 2020). In the sender states, too, economic restrictions may have distributive consequences to the detriment of certain domestic groups (Giumelli, 2017). Hence, scholarship should devote more attention to the conditions under which policy-makers opt for an economic design of sanctions over alternative design choices (see also Bapat et al., 2020: 451).

The EU is a particularly interesting case to explore design choices of sanctions as it has lately opted for broad commercial and financial restrictive measures in addition to targeted restrictions such as asset freezes or travel bans (Giumelli et al., 2020 European Union Sanctions Database (EUSD)). This turn to economic sanctions is puzzling for two reasons. First, one of the EU’s most active fields of external relations is trade. The Union is at the forefront of negotiating free trade agreements in a highly interdependent world of global value chains. Yet, economic sanctions are the exact opposite to free trade agreements since they disrupt rather than generate trade (Hafner-Burton & Montgomery, 2008: 214). Second, economic sanctions often impose costs on the sender states and incur distributive consequences for domestic actors such as business groups (Giumelli, 2017; Pond, 2017). At the same time, the member states of the EU have to agree on restrictive measures, which fall under the Common Foreign and Security Policy (CFSP), by unanimity. Taking unanimous decisions on EU sanctions, the member states will likely push for limited restrictions in order to reduce any potential economic consequences for their domestic actors. For these reasons, economic sanctions by the EU were already considered “relics for the history books” (Hellquist, 2012: 44). Against this background, the question emerges why and under what conditions the EU turns to economic sanctions when designing restrictive measures?

The contribution of this article is threefold. First, by exploring the conditions under which the EU turns to economic sanctions rather than targeted restrictions the study adds to the debate on how actors make foreign policy design choices (e.g. Jupille et al., 2013). Within the realm of EU external relations, CFSP sanctions are one of the most important foreign policy tools. The variation between (EU) economic and targeted restrictions is not the only choice in designing sanctions but arguably an important one as economic sanctions carry domestic costs for the sender (Giumelli, 2017; Pond, 2017) as well as the target (Peksen, 2019). Second, in the scholarly literature on sanctions, design choices have so far remained little examined (Giumelli, 2011; Hedberg, 2018; McLean & Whang, 2014). While the conditions under which states adopt economic sanctions have received much attention (e.g. Hafner-Burton & Montgomery, 2008; von Soest & Wahman, 2015), the choice of economic restrictions over alternative designs has hitherto remained underexplored (see also Bapat et al., 2020; Drezner, 2011: 96). This article fills this lacuna in the literature by systematically investigating the design choice of economic versus targeted restrictions. Third, empirically, the accompanying dataset (online appendix^1^) represents a systematic mapping of EU CFSP sanctions that, among the first ones, includes the dimension of fine-grained design choices among a range of sanctions tools: economic measures including commodity sanctions, dual-use embargoes, and financial restrictions as well as targeted measures including asset freezes, arms embargoes, and travel bans.

The article proceeds by, first, reviewing existing literature on sanctions with a view to the drivers of specific design choices. The second section develops a nuanced classification of sanctions’ designs ranging between economic and targeted. Third, I develop expectations rooted in international- and domestic-level theorizing regarding the conditions under which decision-makers turn to an economic rather than a targeted design of sanctions. In the fourth section, I introduce the data on EU CFSP restrictive measures and the set-theoretic methodological approach. Fifth, the article provides descriptive results on the variation of sanctions’ designs, and, sixth, empirically examines the combinations of conditions that trigger an economic design of restrictive measures. In the conclusion, I summarize the findings and reflect on avenues for further research.

## State of the art

Scholarly literature on international sanctions has a strong focus on their success or effectiveness (e.g. Biersteker et al., 2013; Hufbauer et al., 2007; Seitz & Zazarro, 2020). In addition to effectiveness, most research primarily examines whether and why a sender adopts or does not adopt sanctions, thereby relying on a dichotomous conceptualization of the outcome (Hafner-Burton & Montgomery, 2008; von Soest & Wahman, 2015). Such a narrow conceptualization of the dependent variable overlooks the broad variation senders have when they design sanctions. Indeed, Giumelli (2011: 23) called for “research aiming at explaining the variance of sanctions”. Similarly, Drezner (2011: 96) urged the scholarly community to investigate the “conditions under which different kinds” of sanctions are deployed as did Bapat et al. (2020: 451) in a recent review essay. The recently published EUSD dataset by Giumelli et al. (2020) delivers the most comprehensive data on EU sanctions and their types to date. Far too few studies in the sanctions literature, however, compare and analyze different designs of sanctions (Giumelli et al., 2020; Hedberg, 2018; McLean & Whang, 2014; Kreutz, 2017; Portela, 2016).

One strand in the literature assumes that and tests in how far the EU designs sanctions in such a way as to punish human rights violators (Kreutz, 2017; Portela, 2016). Kreutz (2017) explores the variation between EU foreign policy inaction versus action by taking into account economic sanctions and military interventions. He thereby contextualizes the use of economic sanctions as one design choice among the range of civilian or military missions and complete inaction. The empirical findings demonstrate that human rights violations in a third country are, in fact, an explanatory factor that drives EU action in the form of economic sanctions. Similar to Kreutz (2017), Portela (2016) assumes that sanction senders like the EU seek to punish those who are responsible for humanitarian atrocities. Her study investigates the extent to which the EU designs targeted sanctions in such a way that they harm the responsible elites or individuals across CFSP restrictive measures, aid suspension and trade sanctions. Among these different types of sanctions, the design of CFSP targeted sanctions prove, however, not to reflect the motivation to punish the regime and protect the civilian population of a target state. Hence, both studies start from the assumption that the EU designs sanctions in such a way that they harm human rights violators. Yet, the empirical findings by Kreutz (2017) and Portela (2016) remain inconclusive regarding the extent to which this assumption translates into the design choices of EU restrictive measures.

McLean and Whang (2014) offer a different perspective on the design of sanctions by endorsing a domestic politics explanation. Based on the seminal studies by Kaempfer and Loewenberg (1988, 1999), they argue for a two-stage model of sanction decisions which responds to domestic actors. In a first stage, policy-makers take a decision to adopt sanctions in order to respond to pressure from voters when their country faces a target’s controversial actions. In a second step, these policy-makers take a design decision and choose a specific sanctioning instrument in order to respond to lobbying activities and pressure by domestic interest groups. Hence, this two-stage model expects more limited, targeted sanctions when the sender expects high domestic costs and therefore has to respond to lobby groups within the own country (McLean & Whang, 2014). Likewise, Weber (2019) argues that policy-makers face a trade-off when taking sanction decisions: on the one hand, they want to hurt the targeted regime and impose high costs on them; on the other hand, policy-makers seek to protect their domestic interest groups and shield them from the costs arising from economic sanctions. For this reason, Weber (2019) argues that economic sanctions are less likely against significant trade partners.

Hedberg’s (2018) findings, however, do not confirm such an effect of domestic politics on the design of Russian sanctions against the EU and North American states. She therefore advances an explanation rooted in geopolitical considerations which she terms ‘differentiated retaliation’. More specifically, Hedberg (2018) argues and finds empirically that geostrategic considerations have an impact on a sender’s sanctions decision in such a way that the specific design hurts rival actors (Drezner, 1999) and shields strategically important states. Hence, Russia’s agricultural sanctions of 2014 were designed, by the inclusion or exclusion of specific foodstuff, so as not to harm its strategically most significant partners (Hedberg, 2018: 47).

As the review of the studies above suggest, scholarship on sanctions and their design provides no conclusive explanation as to why and how senders make specific design choices on restrictive measures. While the scholarly community advances explanations of different kinds rooted in normative, human rights considerations (Kreutz, 2017; Portela, 2016), domestic politics (McLean & Whang, 2014; Weber, 2019), or geostrategic motivations (Hedberg, 2018), we still lack strong empirical evidence for either of these three theoretical approaches. A potential reason for this is that a combination of different factors under specific circumstances or even multiple, alternative pathways explain the varying designs of sanctions (Boogaerts, 2018; Boogaerts & Drieskens, 2020; Grauvogel & von Soest, 2014; Portela, 2010; Giumelli, 2011). In fact, there seems to be an accordance in the literature that restrictive measures are context-sensitive and several combinations of explanatory factors trigger the adoption of sanctions (Biersteker et al., 2013: 7). In the following, the article therefore theorizes a set of four conditions stemming from varying theoretical perspectives, including normative, human rights considerations, domestic politics, geopolitical concerns, and diffusion effects, in order to investigate empirically in the subsequent section which of these conditions or combinations of conditions explain different designs of EU sanctions, economic versus targeted. Before doing so, the next section classifies EU restrictive measures based on a nuanced conceptualization of economic versus targeted sanctions’ designs.

## Conceptualizing the design of sanctions

Notwithstanding a flourishing research agenda on sanctions, no clearly defined classifications exist on the different designs of restrictive measures. This applies particularly to the EU where sanctions appear in multiple policy fields, covering development cooperation, international trade, and CFSP (Donno & Neureiter, 2018; Fürrutter, 2019; Hollyer, 2010; Koch, 2015; Meissner, 2021; Portela, 2010). The result is a certain degree of confusion in the literature and in current datasets as to what counts as economic versus targeted sanctions (Biersteker et al., 2020: 5).

In an endeavor to provide a more systematic conceptualization of sanctions’ design, I develop a nuanced classification of restrictions into economic versus targeted based on two dimensions. I adopt this classification with an empirical focus on autonomous EU CFSP restrictive measures under article 215 Treaty on EU.^2^ A first dimension refers to the type of sanctions employed: economic versus non-economic. In line with the literature, I conceive of economic sanctions as an “act by a sender […] to disrupt economic exchange” (Drezner, 2003: 643). Consequently, I consider export and import bans, major financial sanctions or dual-use restrictions as economic sanctions. Travel bans or arms embargoes, by contrast, are considered as non-economic sanctions as they do not fall under the classical category of interrupting regular economic exchange between a sender and a target (similar to Portela & van Soest, 2012: 5).

A second dimension refers to the scope of sanctions whether they are comprehensive or more selective, i.e. targeted. Comprehensive sanctions, as an ideal type, are those that entail costs for the entire population (van Soest & Wahman, 2015: 965) such as bans on all exports to a target state. Targeted sanctions, by contrast, entail costs not for the entire population but only for particular individuals, firms or political entities (Hufbauer et al., 2007: 138; Morgan et al., 2014; van Soest & Wahman, 2015: 965). In the real world, the scope of sanctions is a continuum ranging between targeted measures on a handful of individuals versus a full-on embargo. The latter type, however, is hardly adopted any longer (Biersteker et al., 2016). If we were to understand all sanctions beneath a full-on embargo as a targeted measure, we would miss out on the broad variation of sanctions’ designs. I therefore argue that we need a more nuanced understanding of what counts as a targeted and what counts as a comprehensive measure. A helpful indication for such a nuanced classification is the restrictions’ expected impact on the target population (see also Biersteker et al., 2020). Sectoral sanctions have a significantly higher impact on a target state than non-sectoral ones since they affect an entire branch of the economy like oil or gas with inherent implications for the population at large. I therefore consider sectoral sanctions as comprehensive whereas I classify non-sectoral sanctions as targeted.

Taking these two dimensions – economic versus non-economic and comprehensive versus targeted – together, I adopt the following classification of sanctions’ designs. I only consider those restrictions as economic in design that classify as an economic type and are sectoral in scope: To these belong export and import bans, financial restrictions (other than asset freezes), and dual-use sanctions. Other designs of sanctions are either non-economic in nature like arms embargoes or have a targeted, non-sectoral scope like asset freezes or travel bans. I think of these sanctions’ designs as targeted.

## Theorizing the design of sanctions

In this section, I rely on the sanctions literature (e.g. Giumelli, 2010; Kreutz, 2017; McLean & Whang, 2014; Portela, 2016) which I integrate with international relations theorizing (e.g. Drezner, 1999; Hafner-Burton & Montgomery, 2008; Simmons & Zachary, 2004) in order to develop explanatory conditions for the EU’s design choice of economic versus targeted restrictions. The conditions put forward in this article map explanatory power on a domestic level through the normative motivation of protecting human rights and domestic politics as well as on the international level through geopolitical concerns and diffusion effects in making design decisions (similar to Binder, 2015). Hence, I explore and test which of these conditions, or which constellations of conditions, yield explanatory power for the design decisions on sanctions.

As McLean and Whang (2014) suggest, I think of the design choice in the decision-making on sanctions as a two-stage process. In a first step, states or international organizations decide to adopt sanctions against a specific target state due to a perceived misbehavior. Once actors like the EU have decided to impose a sanction, they need to make a decision about what concrete measures to adopt: economic measures such as commodity embargoes, dual-use sanctions, and financial restrictions or targeted sanctions in the form of asset freezes and travel bans or a combination thereof. This article focuses on the second step regarding senders’ design decisions and concentrates primarily on the conditions under which actors decide to impose economic sanctions in addition to or as an alternative to targeted sanctions.

### Domestic-level theorizing: normative considerations and domestic politics

Much of the literature on sanctions assumes and explores whether sanctions are employed by states or international organizations in order to put a halt to fundamental rights violations in a target country (Hellquist, 2012; Kreutz, 2017; Nielsen, 2013; Portela, 2016). Research on the EU as a sender of sanctions is particularly advanced in testing the extent to which violations of international norms trigger restrictions under the European CFSP (Hellquist, 2012; Kreutz, 2017), in the context of European trade policy (e.g. Saltnes, 2018), or under both umbrellas (e.g. Portela and Orbie, 2014). Theoretically, this research starts from the premise of the EU’s normative power (Manners, 2002), arguing that the EU as an international institution *sui generis* is based on a set of norms and values which it seeks to export to third countries. The EU, according to this perspective, is based on quasi-constitutional norms, namely democracy, the rule of law and human rights (Sjursen, 2006: 244). Because these principles are enshrined in the EU’s set-up it is predisposed “to act in a normative way” (Manners, 2002: 242). CFSP sanctions are a particularly powerful tool to promote certain norms such as human rights and to punish violations thereof in the target state (Hellquist, 2012; Portela, 2016). It is evident in the literature that the EU uses CFSP measures to respond to a violation of norms like human rights (e.g. Hazelzet, 2001; Kreutz, 2017). An unresolved question is, however, under what conditions and with which concrete measures the EU responds to norm violations (Meissner, 2021; Saltnes, 2017; Saltnes & Mos, forthcoming).^3^ Saltnes (2017) puts forward a theoretical approach whereby actors identify norms violation, assess their weight, and evaluate which measures actors then find valid in a concrete situation. The underlying assumption is that the EU adopts proportional sanctions. We can therefore expect that under grave humanitarian atrocities the EU employs hard restrictions and turns to an economic design of sanctions (Binder, 2015; Kreutz, 2017). Empirical research suggests that this is particularly likely in combination with other conditions such as the salience or visibility of human rights violations in the European public and the EU wants to demonstrate action (Binder, 2015; Nielsen, 2013; see below). From the perspective of the EU’s normative power and its motivation to export values such as physical integrity and fundamental rights, severe humanitarian atrocities are therefore a likely condition for the imposition of economic sanctions.

As indicated above, human rights violations are a likely condition to work in combination with the domestic context in triggering a specific design of sanctions. One such context condition rests in domestic politics and focuses on the influence of voters or interest groups when senders design sanctions (e.g. McLean & Whang, 2014; Murdie & Peksen, 2013). An underlying assumption is that policy-makers are responsive to public pressure and have incentives to appease their voters (McLean & Whang, 2014: 593). Hence, they become active and react to a conflict with a specific instrument when the public is highly aware of it. Awareness by the public in political science literature is grasped as the salience of an event (e.g. Ang & Peksen, 2007). Saliency of a conflict can fall together with the threat imposed by a target state on the sender or the intensity of a violent conflict (see sub-section below). However, this is not necessarily the case as the public or media can pay strong attention to an international conflict albeit a low level of security threat, and pressure state-like actors to address these conflicts (Binder, 2015). In these situations, a sender like the EU wants to be perceived as a credible actor in front of the public audience (Hellquist, 2012; see also Nielsen, 2013). Hence, it is likely that the EU designs sanctions in such a way that they are visible to domestic electorates. As economic sanctions affect a large variety of domestic actors within the sender, they can be assumed to be more visible in European public compared to non-economic, targeted sanctions like travel bans.

### International-level theorizing: Geopolitical concerns and diffusion effects

An alternative theoretical perspective compared to domestic politics is rooted in the international system and ascribes explanatory power to factors located on the international level. Indeed, scholarly research on sanctions suggests that geopolitical concerns can drive a sender’s behavior in design choices on restrictive measures (Drezner, 1999; Hedberg, 2018). Among geopolitical considerations is the political or security threat by the target country (Binder, 2015) which has a likely influence on the design of its restrictions. This is related to the assumption that the *objective* of a sanction regime has an impact on the concrete *measures* used. Von Soest and Wahman (2015), for example, argue that concerns about a target’s regime type are likely to result in targeted sanctions. While in situations of considering security objectives, by contrast, senders are more likely to apply ‘hard’ sanctions which constrain or coerce targets (Giumelli, 2010). The assumption behind this argument is that sanctions are ‘proportionate’ and reply to the target’s misbehavior. This can be best achieved when actors apply economic sanctions which impose high costs on the target state (Giumelli, 2010: 38-39; Hedberg, 2018). Economic sanctions like financial restrictions or commodity embargoes cause high material costs for the target state given that these interrupt trade or finance relations with the EU – which is a major power in trade (Drezner, 2007). Hence, EU geopolitical considerations in the form of a political or security threat imposed by the target state is a likely condition for the adoption of economic sanctions.

Besides the target state and threat emanating from it, sender actors might be influenced from third countries, too, when they make design choices on sanctions (see also Martins 1992). International relations theorizing suggests that such an impact happens through diffusion: whereby policy choices of some governments or actors influence the choices made by other states or international organizations (Elkins & Simmons, 2004: 172). Diffusion, in other words, claims that “institutional choices of some actors systematically shape those of other actors” (Lenz 2018: 45) through a set of different mechanisms. In the context of the EU, scholarship documents diffusion effects from Europe to other organizations (e.g. Börzel & Risse﻿, 2012; Lenz & Burilkov, 2017). However, diffusion effects might also occur in the other direction from third countries to the EU whereby design decisions on sanctions are driven by previously adopted regimes outside of Europe. A straightforward example is the implementation of UN sanctions which the EU automatically translates into CFSP decisions. Yet, we might also think of an impact exerted by the US, one of the most important sanctions senders worldwide, according to which EU decision-makers adopt an economic design of restrictions only when the US, too, imposes a strong sanctions program. Indeed, Borzyskowski and Portela (2016) find high levels of cooperation among multiple senders of sanctions. Martins (1992) theorized such effects emanating from the US on actors’ sanctions programs as cooperative endeavors ranging on a scale between coercion and co-adjustment. While it is not vital in the context of this study through which mechanism – coercion, co-adjustment (Martins, 1992), competition, learning or emulation (Simmons et al., 2006) – this effect occurs, diffusion stemming from the US can be theorized as both a coercion mechanism and an emulation mechanism (see also Martins, 1992). According to coercion, the US government would exercise pressure on other actors like the EU to adopt sanctions on a particular country through a manipulation of information or of the costs and benefits in relations between the EU and the target state (Martins, 1992; Simmons et al., 2006: 790). Coercion in the context of sanctions is particularly likely when the US has a strong, asymmetric interest in economic sanctions, when it bears high costs from a sanction program, and therefore exerts pressure, makes promises or imposes a threat on actors like the EU to adopt a similar set of sanctions, too (Martins, 1992: 30). According to an emulation mechanism, by contrast, we might expect that the EU looks at the US as a pioneer in sanctions decisions who sets the benchmark of what is considered an appropriate reaction to a target’s misbehavior (Simmons et al., 2006: 799). Martins (1992: 39) theorized such situations as co-adjustments whereby actors coordinate their action in the belief that a target’s behavior requires restrictive measures and that a specific type of sanction is an appropriate response. Data from elite interviews with decision-makers in the sanctions realm indicate coercion and co-adjustment effects. According to this data, decision-makers in the EU and member states have to respond to pressure exerted by the US (Interview 1, 2019; Interview 2, 2019) and they adapt sanctions’ design with a view to information retrieved from the US (background talk). To the best of my knowledge, diffusion effects have, to date, not been tested in empirical research on sanctions decisions. Yet, it is plausible from a theoretical and from an empirical perspective to expect that such an effect occurs on the EU, especially when it makes a design decision in favor of economic sanctions, once the US launches a similar sanctions program. Hence, I expect the EU to adopt economic sanctions when the US does so, too.

In brief, I expect sender actors like the EU to adopt an economic design of sanctions under conditions of domestic- and international-level relevance: (a) when the target commits grave human rights violations, (b) when the conflict is salient in public, (c) when the target imposes a political or security threat on the sender(s), and when (d) the US imposes a sanctions program on the same target. While all of these factors are conditions under which an economic design of restrictions may be adopted, I expect a constellation of conditions rather than a single condition, and multiple pathways rather than a single explanation as explanatory towards the outcome. In other words, I expect design choices on sanctions to be context-sensitive and that several combinations of explanatory factors may trigger the outcome (Schneider & Wagemann, 2012). In the subsequent section, I therefore elaborate on the set-theoretic methods strategy I pursue in this study and how I measure (calibrate in set-theoretic jargon) the conditions and the outcome in the context of this research article.

## Set-theoretic methods, calibration of conditions, and data sources

Against the backdrop that restrictive measures are context-sensitive and several combinations of explanatory factors may trigger the adoption of sanctions, the application of set-theoretic methods appears to be an appropriate choice. More specifically, I employ crisp-set Qualitative Comparative Analysis (QCA) using the QCA (Dusa, 2019) and the SetMethods packages (Oana & Schneider, 2018) in the software R. The choice of QCA is motivated by the assumption that a combination of different conditions and multiple pathways trigger design choices regarding economic sanctions vis-à-vis targeted sanctions. Based on these assumptions of conjunctural causation and equifinality (Schneider & Wagemann, 2012), QCA, including its crisp-set version, has already been applied successfully to the study of EU sanctions (Grauvogel & von Soest, 2014; Giumelli, 2010; Portela, 2010). I use the crisp-set rather than the fuzzy-set or multi-value version of QCA for two reasons. First, I am primarily interested in when, the conditions under which, the EU employs an embargo in the form of economic sanctions rather than the gradual aspect of how many persons or entities are targeted. Second, given the limited amount of restrictive measures currently in force in which the EU turns to economic sanctions (six in total), the data for the outcome cannot be transformed into a meaningful fuzzy-set calibration.

The universe of cases exists of EU sanctions regimes in force. This is due to the article’s primary interest in sanctions’ design rather than the question *whether* the EU adopts restrictions. Out of the universe of 43 EU sanction regimes in force in 2019, I select 19 cases for the QCA analysis. One reason for choosing a subset of cases is the deletion of horizontal sanction regimes such as the measures to combat cyber-attacks or terrorism and the deletion of UN sanctions which the EU implements while having made no design choice. The latter types of sanction regimes were excluded from the dataset for the QCA analysis because they would not allow for a sensible empirical investigation into the drivers of EU design decisions. The horizontal sanction regimes are not part of the empirical analysis, because the conditions outlined in the theoretical section operate on the level of countries and cannot be measured for terrorist groups or cyber-attacks. Moreover, I chose to exclude those cases where data on the conditions was missing partly because CFSP restrictive measures are still in force in 2019 but date to pre-Lisbon decisions. These countries include China (1989), Haiti, Libya, Montenegro and Serbia (all 1990ies). Furthermore, I deleted the EU sanctions on the US because these concern primarily the protection of European industry from US sanctions’ extraterritorial application and are therefore driven by a rationale different than restrictive measures against countries which commit human rights violations or breaches of international law. Out of the 19 remaining sanction cases, the EU opted for an economic design of sanctions in six cases. Given the N=19, I have to restrict the QCA analysis to four conditions (N must be at least 2^k^ where k constitutes the number of conditions).

In order to calibrate the conditions and the outcome according to a crisp-set QCA, I rely on a dichotomous calibration (the online appendix summarizes the set membership of cases in the conditions and the outcome). Where appropriate, I choose a qualitative data-driven strategy (De Block & Vis, 2018). In an online appendix, I report sensitivity tests and robustness checks regarding alternative conditions and alternative ways of calibrating the conditions based on the raw data.

The outcome, i.e. economic sanctions (ECON), was coded 1 when the EU employed financial restrictions other than pure asset freezes, dual-use restrictions, or sectoral sanctions on commodities or services or both; ECON was coded 0 when the EU opted for targeted measures or an arms embargo. The outcome ECON appeared in six out of the 19 cases, i.e. in six country cases the EU adopted sectoral sanctions on commodities or services, dual-use goods, or financial restrictions that go beyond an asset freeze.

Regarding fundamental rights violations and the severity of a conflict, I rely on the proxy of state-based violence and the number of related deaths in the target country, similar to previous studies (Binder, 2015; Kreutz, 2017). In order to measure the deaths arising out of state-based violence (FAT), I draw on the Uppsala Conflict Data Program (Gleditsch et al., 2002; Pettersson & Öberg, 2020). When a target state committed violence on its citizens resulting in deaths during the period under observation, FAT was coded 1. When no fatalities were incurred by state-based violence, FAT was coded 0.

In order to measure the saliency of a conflict (SAL) I rely on the public visibility and media coverage (Warntjen, 2012). More specifically, I ran a guided news search and counted all newspaper articles on the country and its conflict in the Guardian employing LexisNexis University. More specifically, the news search was based on the targeted country and the concrete issue of the conflict, e.g. ‘Burundi, Coup’ or ‘Russia, Crimea, Annexation’. I collected the data on a yearly basis starting from one year preceding the first sanction decision and calculated the average yearly media reporting in the Guardian when decisions were taken in more than one year. Data on media attention were collected only for the time period preceding a sanctions decision. Based on the raw data, I consider the saliency of a country conflict as high when the Guardian reported about it at least 100 times, in which case SAL was coded a 1. In cases where the Guardian reported less than 100 times, SAL was coded 0.

With a view to the political or security threat imposed by a country, I rely on the Correlates of War National Material Capabilities data (Singer et al., 1972). More specifically, I measured the condition capabilities (CAP) based on the military personnel of a country in the year of a sanctions decision or in the year with the latest information available (2012 in the dataset). The online appendix reports sensitivity tests when using a measurement other than the military capabilities of a country using the condition THREAT. With a view to threat (THREAT), I use a combined score of the target’s geographical distance in kilometers from Brussels where the EU headquarters reside and the military strength of a country measured by the data for military expenditure in constant price (2017) in millions US Dollar (SIPRI, 2020).

Regarding the measurement of US sanctions (USE), I drew on data from the US department of the treasury, which reports all US American sanctions programs. I cross-checked this data with the EUSANCT dataset (Weber & Schneider, 2020). Where an economic sanction program on the country in the form of a (partial) economic embargo, an import or export restriction, a commodity sanction or a major financial sanction was reported, I coded USE a 1. In the absence of an economic sanction program, i.e. a targeted financial sanction, a travel ban or an arms embargo, USE was coded 0. The measurement of US sanctions programs coded as 1 includes only those programs that preceded or entered into force at least in the same year as an EU restrictive measure.

## The design of sanctions: Descriptive empirical results

To capture the design of EU sanctions, I have collected data on CFSP autonomous restrictive measures that were in force in 2019. The reason for focusing on the EU’s autonomous sanctions is that it has a free choice in designing the specific instruments. Whereas in the case of implemented UN sanctions the EU simply transfers those programs to the European level.

**Fig. 1:**
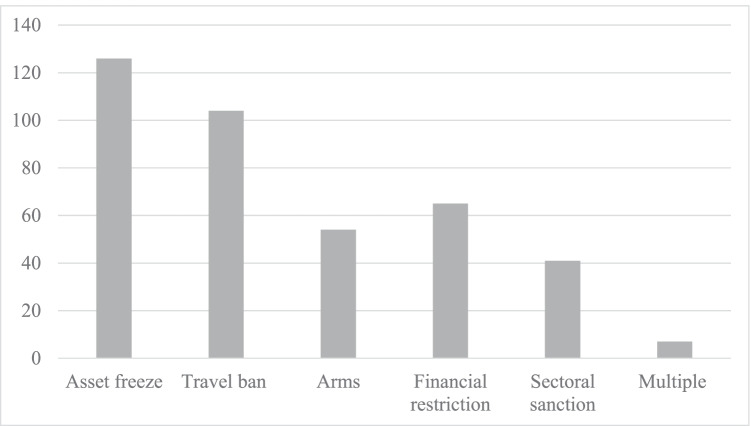
The design of EU decisions on restrictive measures (in force in 2019). Source: own illustration

A first empirical observation is that EU sanctions have significantly increased in number since the Maastricht Treaty entered into force (Giumelli et al., 2020: 9). While before the 2000 s the amount of CFSP sanctions regimes were at a moderate amount of below ten, we observe a boost in 2011 due to the events in the Middle East and North Africa (MENA) region and a steady increase of sanctions regimes after that year (Giumelli et al., 2020: 9). At the same time, Weber and Schneider (2020: 11) in their recently released EUSANCT dataset find that EU sanctions are highly successful at a positive rate of about two-thirds. Based on these observations, EU CFSP sanctions are no trivial instrument and have a real-world impact on third countries. It is all the more important to investigate how – with which designs – the EU sanctions its targets. Over time, the EU has proven to be a relatively coherent sanctioning actor regarding the design of restrictive measures adopted (Giumelli et al., 2020: 16; Fig. 3 in online appendix). Asset freezes and travel bans have increased in absolute numbers as did the total amount of EU sanctions. Earlier studies and data gathering efforts find these types of sanctions the most frequent to occur at 75% and 62% respectively between 1989 and 2019 (Giumelli et al., 2020). These numbers are confirmed by the EU’s current primary use of targeted sanctions on individuals through asset freezes (14 sanction cases) or travel bans (12 sanction cases) (Fig. 1). Over time, the EU has also made increasing use of arms embargoes and commodity sanctions, alongside a continuing employment of major financial restrictions (Giumelli et al., 2020: 15). The majority of the EU’s commodity embargoes and financial restrictions have been employed in the last nine years: out of 14 commodity and 14 financial restrictions, 11 and eight respectively have been imposed by the EU since 2010. These data reinforce the evaluation by scholars like Clara Portela (2016) that the EU has lately employed more comprehensive sanction regimes. This is corroborated by the EU’s current usage of comprehensive sanctions as it has in force financial restrictions that go beyond asset freezes in three cases, sectoral sanctions on arms eight sanction cases, and, most strikingly, sectoral sanctions on commodities or services imposed in six sanction cases. Compared to the period between the Maastricht Treaty and 2019, the relative amount of economic sanctions such as in the form of commodity embargoes has therefore slightly gone up from 18% to about 26% (Giumelli et al., 2020). The absolute *and* relative increase of economic sanctions, and the exceptionally high amount of such restrictions in force in 2019 makes a study of the EU’s design choices of its restrictive measures in the respective year – 2019 – particularly interesting. Hence, while overall the majority of sanctioning decisions follows a targeted format, it are especially the design choices of financial restrictions or sectoral sanctions on commodities or services, which deserve closer investigation in this article.

When exploring the EU’s economic sanctions further, financial restrictions (those other than asset freezes) seem to appear together with sectoral bans on commodities or services in the cases of Russia, Syria, and the Ukraine, more concretely Crimea and Sevastopol. Indeed, CFSP restrictive measures on Russia, Syria and the Ukraine (Crimea and Sevastopol) are the most comprehensive sanctions regimes that the EU currently has in force. On these targets, the EU imposed broad financial restrictions regarding the trade of bonds, fulfillment of contracts or transactions, investment services, loans or credit (Russia, Syria, Crimea and Sevastopol) and the acquisition of real estate (Crimea and Sevastopol) in addition to commodity and services bans (EU sanctions map 2020). In addition to these three countries, the EU has a further set of sectoral sanctions on commodities, dual-use goods or services in force against Iran, Myanmar/Burma, and Venezuela.

The frequent use of economic sanctions is not unique to the EU. We observe financial restrictions and sectoral commodity or dual-use bans in sanctions regimes of other actors, too (Fig. 2 in online appendix). Similar to the EU, the UN and also the US primarily employ targeted sanctions imposed on domestic entities through travel bans (22 in total in case of the UN; 25 in total in case of the US) or asset freezes (28 in total in case of the UN; 23 in total in case of the US). However, as is the case with the EU, the data show a substantial amount of sectoral UN and US sanctions, especially in the area of arms exports and imports (employed in 35 UN sanctions episodes and 30 US sanctions programs). Moreover, the UN imposes sectoral sanctions in the area of finances and in the area of commodities as does the US: in three UN sanctions episodes, sectoral financial restrictions other than individual asset freezes were employed by the UN in addition to 23 commodity or services sanctions. The US imposed financial restrictions and commodity and services sanctions in 16 cases each. In current scholarship, this variation of economic sanctions such as financial, dual-use or commodity restrictions versus other sanctions instruments like travel bans or asset freezes has yet remained underexplored.

## Analyzing design decisions on international sanctions

In this section, I turn to the empirical analysis of how senders like the EU make design decisions on international sanctions and the conditions under which they opt for economic restrictive measures rather than targeted ones.

When exploring the variation between economic and non-economic sanctions, the first step in the QCA analysis is a test for necessary conditions based on parameters of fit that are the consistency, coverage, relevance of necessity (RoN) scores. The online appendix reports the results of the analysis of necessity across the 19 cases and for the combination of all four conditions, i.e. fatalities arising out of state-based violence (FAT), saliency of a conflict (SAL), military capabilities (CAP), and the imposition of US economic sanctions (USE). According to the empirical analysis, no condition passes the consistency threshold of 1.000 which is recommended in the crisp-set version of QCA. This means that none of the conditions can be considered necessary for the EU to adopt economic sanctions.

In a second step of the QCA analysis, I create and minimize a truth table in order to investigate sufficient conditions or a combination therefore in explaining the EU’s design choices in favor of economic sanctions. The truth table for the set of EU sanctions (online appendix) displays nine rows with empirical cases omitting the logical remainders, but including the parameters of fit consistency and proportional reduction in inconsistency (PRI), the outcome, the number of cases, and the constellation of conditions military capabilities (CAP), fatalities (FAT), saliency (SAL), US sanctions (USE). When creating the truth table, we observe a maximum consistency at a threshold of 1.000 in the rows producing the outcome ECON (online appendix). This implies that none of the cases in a truth table violates the sufficiency relationship. In other words, throughout the procedure of minimizing the truth table, the cut-off point for inclusion is set at a conservative consistency threshold of 1.000. No sanctions case may, thus, violate the sufficiency relationship in a truth table row when including this row in the QCA minimization procedure.

The minimization procedure of the truth table leads to three paths in the conservative solution term, i.e. three possible pathways towards an economic design of EU restrictive measures. The online appendix reports the pathways, their parameters of fit, and the respective EU sanctions cases. Given that I set the cut-off point for inclusion in the minimization procedure at a threshold of 1.000 each of the pathways scores at a maximum consistency and PRI value of 1.000, while the overall coverage score of the conservative solution term is at a maximum of 1.000. This means that none of the country cases violate the sufficiency relationship. Furthermore, the combination of the three pathways, i.e. the conservative solution term, covers the entire set of positive cases in which the EU adopted economic sanctions: the sanction regimes imposed on Iran, Myanmar/Burma in 2018, Russia, Syria, Ukraine3 (Crimea and Sevastopol), and Venezuela.

The combination of military capabilities (CAP) and humanitarian atrocities (FAT) is present in the first two pathways of the conservative solution term. Interpreting this combination empirically, we may think of it as a constellation of a serious misbehavior perceived by the EU whereby a target commits human rights violations and has the actual military capabilities to pursue them. This interpretation would suggest that the EU sanctions countries economically when the regimes violate fundamental rights severely, leading to conflict-related deaths, and when, at the same time, they have the military capabilities to continue doing so and exercising a potential threat to other actors, too. This finding is in line with earlier research that observed a correlation between EU action and human rights violations in combination with geostrategic concerns (Kreutz, 2017, 214). However, the EU does not automatically impose tough, economic sanctions on targets committing fundamental rights violations that have strong military capabilities. Rather, US sanctions (USE; first pathway) or the saliency of a conflict (SAL; second pathway) have to accompany a target’s serious misbehavior in order for the EU to impose economic sanctions. The first pathway of the solution term combines the threat imposed by the targeted third country, humanitarian atrocities, and the presence of US sanctions: CAP*USE*FAT. According to this pathway, the effect of accompanying US sanctions in combination with humanitarian atrocities and a country’s military capabilities is sufficient for the EU to impose economic sanctions. This may be interpreted in such a way that the pathway covers those cases that pose a major political or security threat to the EU, where the country actually uses its military potential as evident in form of casualties, and where the EU can ally with other actors like the US when adopting an economic sanctions regime. Two cases, the EU sanctions against Russia and the Ukraine3, namely Crimea and Sevastopol, are uniquely covered by this pathway. Iran and Syria are covered by the pathway, too, however, not uniquely. From a theoretical perspective, the pathway is plausible as we know from scholarly research on sanctions that multilateral coalitions of restrictive measures make the latter more likely prone to success (Bapat & Morgan, 2009; Early & Spice, 2015; Weber & Schneider, 2020). Hence, the EU may take the potential support by further actors like the US into account when making design decisions on economic sanctions regimes.

Indeed, fine-grained information on the EU’s tough sanctions package imposed on Russia confirms the pathway as identified by the QCA analysis. Restrictive measures against Russia, and as a corollary against Crimea and Sevastopol, were adopted in three steps in 2014 and increasingly escalated into economic restrictions in the dual-use and oil sectors as well as regarding technology and software (Council Decision 2014/512/CFSP). Tough, economic sanctions were eventually adopted despite initial reluctance by some of the EU member states like Austria, Italy, or Hungary (Giumelli, 2017; Portela et al., 2020) upon the downing of MH-17 and under the lead of Germany (Schoeller, 2020). Scholars show how the downing of the passenger flight MH-17 and the resulting casualties were a game changer and made EU member states, especially Germany, perceive Russia and the Ukraine crisis as a major political and security threat potentially disrupting international relations in Europe (Schoeller, 2020; Speck, 2016; Szabo, 2014). However, while the perceived threat imposed by Russia and the fear of a further use of its military capabilities were an important driver for Germany to push its European partners for economic restrictions, the backing of the US was a vital trigger for the eventual adoption of tough sanctions (Mearsheimer, 2014; Speck, 2016). Speck (2016: 8 ff.) traces the exact decisions taken by the EU and the US and demonstrates how European leaders followed suit whenever the Obama administration took action: in several steps, the EU even met on the same day when the US announced further sanctions and agreed on similar restrictions. After the downing of MH-17, the EU and the US decided in tandem upon a video conference to escalate the sanctions to sectoral, economic ones (Speck, 2016). Importantly, it was the US which exercised pressure and “very much encouraged” (Embassy of the US, Kyiv, Ukraine 2014 as cited by Speck, 2016: 9) the EU to move to the full, economic sanctions package against Russia (Speck, 2016: 9). Hence, the EU imposed economic sanctions against Russia in view of the target’s political and security threat to Europe combined with the casualties arising from the Ukraine crisis and the downing of MH-17. However, as identified by the first pathway of the QCA analysis, it only did so when it had the backing of the US as an ally pressuring for tough restrictions.

The second pathway of the solution term rests on a country’s and its conflict’s saliency in public in combination with the target’s military capabilities and fundamental rights violations: CAP*FAT*SAL. According to the conservative solution term of the QCA analysis, a conflict’s saliency (SAL) is a sufficient condition for the EU to impose an economic sanctions regime on a country in combination with a level of threat and severe human rights violations. Thus saliency, together with a country’s strong military capabilities and humanitarian atrocities triggers an economic design of sanctions. This means that in light of a country’s military capabilities and severe human rights violations in combination with a country’s conflict saliency in the media and in public the EU eventually turns to tough economic sanctions. In the set of EU restrictive measures, the CFSP economic sanctions on Myanmar/Burma2 (2018) are uniquely covered by this pathway, and Iran and Syria belong are covered by it, too.

In fact, the EU imposed CFSP restrictive measures on Myanmar/Burma in various decisions and alongside restrictions in the areas of development cooperation and trade (Meissner, 2021). Already in 1996, the EU launched restrictions in the form of an arms embargo, travel restrictions, and diplomatic measures on Myanmar/Burma in view of the regime’s violation of human rights and repression of civil society (EU sanctions map, 2020).^4^ In 2018, EU leaders extended the restrictive measures to cover an export and services ban on arm and equipment which can be used for internal repression in addition to financial sanctions, a travel ban, and a prohibition of cooperation and training with the Myanmar Armed Forces and the Border Guard Members (Council Regulation (EU) 2018/647). In the case of Myanmar/Burma, the EU turned to economic sanctions as a response to the Rohingya crisis and the respective humanitarian atrocities (Meissner, 2021). Member states and EU institutions considered the Rohingya crisis “exceptionally serious” (OPP, 2018) and considered the fundamental rights violations against the Rohingya minority in Myanmar/Burma as crossing red lines and a serious breach of international law (OPP, 2018). Myanmar/Burma’s human rights violations happened in a target which has strong military potential: in the set of cases under scrutiny in this article, the country’s military spending comes fourth after Russia, Iran, and the Ukraine (raw data in online appendix). The regime’s military capabilities (CAP) according to the Correlates of War National Material Capabilities data (Singer et al., 1972), too, ascribes to the country fourth rank behind Russia, Iran, and Egypt (raw data in online appendix). Other than in the first pathway and the case of Russia, Myanmar/Burma does arguably not impose an immediate security threat on the EU. However, it has significant military capabilities which can be perceived as a potential threat in the country and the region. The fact that the EU put a halt to military cooperation and training of the Myanmar Armed Forces under the umbrella of its restrictive measures confirms the assessment that Myanmar/Burma is a regime with strong military capabilities and was considered as such by the EU. In addition, the EU expanded sanctions against the country’s military leadership shortly after the coup in February 2021, thereby condemning the “junta’s increasing brutality” (Joseph Borrell as cited by von der Burchard, 2019).Indeed, the second pathway of the QCA analysis identifies the military capabilities and the human rights violations committed by a country as relevant for the EU to take action in form of economic sanctions. Yet, as established in the QCA analysis, European leaders only impose economic sanctions when this constellation of conditions, military capabilities (CAP) and humanitarian atrocities (FAT), is combined with the public saliency of a conflict (SAL). Regarding the case of Myanmar/Burma, public visibility of the Rohingya crisis was exceptionally high and the fundamental rights violations received strong attention within the EU decision-making apparatus (Meissner, 2021). Already since 1991, Myanmar/Burma and its fundamental rights situation has been given attention by EU decision-makers when the European Commission and EU member states imposed pressure on the country through its inter-regional dialogue with the Association of South East Asian Nations (ASEAN), the Council imposed CFSP sanctions, and withdrew tariff preferences in 1997 (Schembera, 2016). Myanmar/Burma became particularly salient in public when in August 2017 more than 600.000 persons of the Rohingya minority fled the country to Bangladesh upon which the EU felt pressured to act (CNN, 2019). Hence, the combination of the human rights violations committed by Myanmar/Burma’s strong military regime against the Rohingya minority in combination with the long-lasting attention devoted to the country by EU decision-makers and the public visibility of the Rohingya crisis made the EU turn to tough, economic sanctions.

The third pathway of the conservative solution term covers only one case where the EU imposed economic sanctions namely Venezuela. Given the pathway’s coverage of only one case, I refrain from interpreting this path substantively. However, the QCA analysis allows the researcher to simplify the conservative solution term by letting the relevant packages in the software R include simplifying assumptions based on the logical remainders through the parsimonious solution term and by ruling out untenable assumptions in the minimization of the truth table through the intermediate solution term. Both the parsimonious and the intermediate solution terms lead to two pathways at maximum coverage, consistency and PRI scores of 1.000 each. Venezuela is covered by the first pathway which is identical in both the parsimonious and the intermediate solution term. The pathway identifies the presence of US economic sanctions (USE) as a sufficient condition for the EU to impose economic sanctions, too. This reinforces the relevance of US sanctions as a condition for the EU to respond with tough, economic measures. The sequence of sanctions’ imposition by the US and the EU indeed suggests a diffusion effect whereby the EU followed suit to the US’s intensified sanctions program. While the US has imposed sanctions for over 15 years, it was during the Trump presidency when sanctions were increasingly tightened and expanded to sectoral, economic sanctions (Congressional Research Service, 2021). In particular, the US sought to exercise pressure on the Venezuelan Maduro regime and its authoritarianism (Congressional Research Service, 2021; Rendon, 2019). A significant expansion of US American sanctions to including sectoral, economic restrictions and major financial measures set in with an executive order in August 2017 (Rendon, 2019). Soon afterwards in November 2017, and also in response to Maduro’s authoritarian regime, the EU tightened its restrictive measures, too, and expanded sanctions to the dual-use sector (Council Decision 2017/2074 of 13 November 2017). To be sure, this is only indicative of a potential US impact on EU sanctions’ design. Still, public commentary on the Venezuelan sanctions, in fact, suggests US American pressure on the EU to broaden and intensify its restrictive measures (e.g. Arostegui, 2019; Spinetto, 2021).

## Conclusions

The EU, in addition to the UN and the US, is one of the most active senders of sanctions and it has steadily increased the quantity of sanctions regimes imposed on third countries (Giumelli et al., 2020). At the same time, EU CFSP restrictive measures are highly successful at triggering a change in the behavior of a target country as recent data shows (Weber & Schneider, 2020). Among the range of European autonomous sanctions, the EU has lately opted more and more frequently for an economic design, employing commodity or dual-use embargoes or financial restrictions or all three, when responding to a target’s perceived misbehavior as the empirics in this article have shown.

In order to investigate the EU’s design choices when it imposes sanctions, I developed a theoretical perspective by combining domestic and international level approaches from which I derived two sets of a total of four conditions: domestic-level conditions that are grave human rights violations and the salience of a conflict as well as international-level conditions that are the threat imposed by a target state and the US as a pioneering sanctioning sender. The empirical findings of the QCA analysis, with the exception of Venezuela, suggest that the EU responds with tough, economic sanctions, as compared to targeted restrictions, to a serious misbehavior: when a target with strong military capabilities commits human rights violations. However, a target’s serious misbehavior triggers EU economic sanctions only when either the US imposes economic sanctions, too, and pressures European leaders into strong activity (path 1) or when a target’s conflict is highly salient in public (path 2). Two pathways therefore explain an economic design of sanctions adopted by the EU. First, the combination of a threat imposed by a target country, the casualties arising from a conflict, and a backing for strong sanctions by the US appears as one pathway for the EU to make a design choice in favor of economic sanctions (path 1). The case of EU sanctions on Russia and the Ukraine, Crimea and Sevastopol, is uniquely covered by this pathway, and fine-grained information confirms the pressure exercised by the US on European leaders to adopted economic sanctions upon the downing of MH-17. The case of Venezuela reinforces the importance of US American sanctions and their pioneering effect on the EU. While Venezuela deviates from the combination of military capabilities and humanitarian atrocities, it belongs to the set of US economic sanctions. Indeed, the EU’s design choice in favor of a dual-use embargo was preceded by a significant expansion of US economic sanctions under the Trump presidency. Second, the saliency of a conflict such as the Rohingya crisis in Myanmar/Burma and the public attention devoted to that crisis triggered the adopted of EU economic sanctions in reaction to the militarily strong regime’s fundamental rights violation against the Rohingya minority (path 2).

This study serves as a starting point for systematically investigating design decisions on sanctions. The variation between economic sanctions, on the one hand, and targeted restrictions, on the other hand, is only one dimension when decision-makers opt for design choices once they adopt such measures. Arguably, other dimensions in design choices like the inclusion or exclusion of specific entities or individuals from sanctions regimes, temporal decisions, or sectoral and product-specific exceptions have so far remained underexplored. This article therefore sees three primary needs for further research. First, further design dimensions, especially the product- and sector-related breadth of sanctions programs and the respective exceptions, require scholarly investigation as so far close to no knowledge exists on which exact companies, products, or sectors are covered by a restrictive measure. Second, this article identifies pathways which explain an economic design of EU sanctions. An intuitive and promising avenue for further research would be to assess the external validity of these pathways by systematically investigating design decisions of other sanctions senders. Third, I theorized a strong US American impact on EU sanctions decisions operating on diffusion effects. The empirical results indeed confirm a strong impact by the US on the EU. To the best of my knowledge, such diffusion effects remain an underexplored terrain in the sanctions literature. Based on the information available in this research article, the mechanisms according to which US decisions diffuse to the EU and its respective design choices on sanctions seem to differ between the Russian and the Venezuelan case. The former resembles a rather coercive US American pressure and the latter appears more like an emulation effect. A promising avenue for further research would be a systematic study, employing rigorous process-tracing methods, of the concrete diffusion mechanisms regarding sanctions’ (design) decisions.

## Supplementary Information

ESM 1 (R 7 kb)

ESM 2 (DOCX 38 kb)

ESM 3 (XLSX 12 kb)

## Data Availability

The dataset generated and analyzed in this article is included in this published article and its supplementary files.

## Footnotes

1 The online appendix is available at the Review of International Organizations’ webpage.

2 I focus on CFSP restrictive measures and exclude EU sanctions adopted in other policy fields like development cooperation or trade due to methodological concerns. More specifically, I seek to hold the institutional rules of decision-making constant which differ tremendously between development cooperation and trade, on the one hand, and CFSP, on the other hand.

3 In cases of nuclear proliferation, weapons of mass destruction, or civil war the EU largely relies on the UN and implements and supplements UN sanctions programs. EU autonomous CFSP restrictive measures rather occur in response to human rights violations or violations of democracy and the rule of law.

4 In 2013, the restrictive measures against Myanmar/Burma were lifted except for the arms embargo and respective services ban (EU sanctions map 2020).
