# Supplementary material for: How to sanction international wrongdoing? The design of EU restrictive measures
Source: Rev Int Organ. 2022 Feb 24;18(1):61–85. doi: 10.1007/s11558-022-09458-0 (PMC9800347; doi:10.1007/s11558-022-09458-0)
Supplement: Supplementary file 2 — (DOCX 38 kb) [file 11558_2022_9458_MOESM2_ESM.docx]

**How to sanction international wrongdoing? The design of EU restrictive measures**

*Katharina L. Meissner*

Online appendix

Accompanying tables pg. 2

Sensitivity tests pg. 6

Accompanying figures pg. 9

Accompanying tables

Table 1: Analysis of necessary conditions for EU economic sanctions

| **EU sanctions** | | | | | | | |
| --- | --- | --- | --- | --- | --- | --- | --- |
| *Condition* | *Consistency* | *Coverage* | *RoN* | *Condition* | *Consistency* | *Coverage* | *RoN* |
| CAP | 0.833 | 0.714 | 0.857 | ~CAP | 0.167 | 0.083 | 0.389 |
| FAT | 0.833 | 0.556 | 0.714 | ~FAT | 0.167 | 0.100 | 0.500 |
| USE | 0.833 | 1.000 | 1.000 | ~USE | 0.167 | 0.071 | 0.278 |
| SAL | 0.500 | 1.000 | 1.000 | ~SAL | 0.500 | 0.188 | 0.188 |

*Source: own illustration*

Table 2: Set membership of cases in the conditions and the outcome

| **FAT** | **CAP** | **SAL** | **USE** | **ECON** | **Case** |
| --- | --- | --- | --- | --- | --- |
| 0 | 0 | 0 | 0 | 0 | Belarus |
| 0 | 0 | 0 | 0 | 0 | Bosnia and Herzegovina |
| 1 | 0 | 0 | 0 | 0 | Burundi |
| 0 | 1 | 0 | 0 | 0 | Egypt |
| 0 | 0 | 0 | 0 | 0 | Guinea |
| 1 | 1 | 1 | 1 | 1 | Iran |
| 0 | 0 | 0 | 0 | 0 | Moldova |
| 1 | 1 | 0 | 0 | 0 | Myanmar/Burma1 |
| 1 | 1 | 1 | 0 | 1 | Myanmar/Burma2 |
| 0 | 0 | 0 | 0 | 0 | Nicaragua |
| 1 | 1 | 0 | 1 | 1 | Russia |
| 1 | 1 | 1 | 1 | 1 | Syria |
| 0 | 0 | 0 | 0 | 0 | Tunisia |
| 1 | 0 | 0 | 0 | 0 | Ukraine (Misappropriation of state funds) |
| 1 | 0 | 0 | 0 | 0 | Ukraine |
| 1 | 1 | 0 | 1 | 1 | Ukraine (Crimea and Sevastopol) |
| 0 | 0 | 0 | 1 | 1 | Venezuela |
| 0 | 0 | 0 | 0 | 0 | Zimbabwe1 |
| 0 | 0 | 0 | 0 | 0 | Zimbabwe2 |

*Source: own illustration*

Table 3: Truth table for the outcome of EU economic sanctions

| *Conditions* | | | | *Outcome* | *N* | *Consistency* | *PRI* | *Cases* |
| --- | --- | --- | --- | --- | --- | --- | --- | --- |
| **CAP** | **USE** | **FAT** | **SAL** |  |  |  |  |  |
| 1 | 1 | 1 | 0 | 1 | 2 | 1.000 | 1.000 | **Russia,**  **Ukraine3** |
| 1 | 1 | 1 | 1 | 1 | 2 | 1.000 | 1.000 | **Iran,**  **Syria** |
| 0 | 1 | 0 | 0 | 1 | 1 | 1.000 | 1.000 | **Venezuela** |
| 1 | 0 | 1 | 1 | 1 | 1 | 1.000 | 1.000 | **Myanmar/Burma2** |
| 0 | 0 | 0 | 0 | 0 | 8 | 0.000 | 0.000 | Belarus, Bosnia and Herzegovina, Guinea, Moldova, Nicaragua, Tunisia, Zimbabwe1, Zimbabwe2 |
| 0 | 0 | 1 | 0 | 0 | 3 | 0.000 | 0.000 | Burundi, Ukraine, Ukraine2 |
| 1 | 0 | 0 | 0 | 0 | 1 | 0.000 | 0.000 | Egypt |
| 1 | 0 | 1 | 0 | 0 | 1 | 0.000 | 0.000 | Myanmar/Burma1 |

*Source: own illustration; positive cases of EU economic sanctions in bold*

Table 4: Conservative solution term for an EU economic sanction design

| *Pathway* | *Consistency* | *PRI* | *Coverage* | *Unique coverage* | *Cases* | |
| --- | --- | --- | --- | --- | --- | --- |
| CAP*USE*FAT | 1.000 | 1.000 | 0.667 | 0.333 | **Russia, Ukraine3** | **Iran, Syria** |
| CAP*FAT*SAL | 1.000 | 1.000 | 0.500 | 0.167 | **Myanmar/**  **Burma2** |  |
| ~CAP*USE*  ~FAT*~SAL | 1.000 | 1.000 | 0.167 | 0.167 | **Venezuela** |  |
|  | 1.000 | 1.000 | 1.000 |  |  |  |

*Source: own illustration*

Table 5: Intermediate solution term for an EU economic sanction design

| *Pathway* | *Consistency* | *PRI* | *Coverage* | *Unique coverage* | *Cases* | |
| --- | --- | --- | --- | --- | --- | --- |
| USE | 1.000 | 1.000 | 0.833 | 0.500 | **Russia, Ukraine3, Venezuela** | **Iran, Syria** |
| CAP*FAT*SAL | 1.000 | 1.000 | 0.500 | 0.167 | **Myanmar/Burma2** |  |
|  | 1.000 | 1.000 | 1.000 |  |  |  |

*Source: own illustration*

Table 6: Parsimonious solution term for an EU economic sanction design

| *Pathway* | *Consistency* | *PRI* | *Coverage* | *Unique coverage* | *Cases* | |
| --- | --- | --- | --- | --- | --- | --- |
| USE | 1.000 | 1.000 | 0.833 | 0.500 | **Russia, Ukraine3, Venezuela** | **Iran, Syria** |
| SAL | 1.000 | 1.000 | 0.500 | 0.167 | **Myanmar/Burma2** |  |
|  | 1.000 | 1.000 | 1.000 |  |  |  |

*Source: own illustration*

Sensitivity tests

*Alternative measures of concepts*

In order to face the potential problem of measurement errors, I report sensitivity tests regarding the measurement and calibration of the conditions as included in the empirical analysis. The measurement of US economic sanctions programs is straightforward, which is why robustness checks are not relevant to the condition USE. The measurement of grave human rights violations through the number of casualties relies on earlier studies which use similar proxies and similar data (e.g. Binder 2015; Kreutz 2017). Hence, I consider the measurement through deaths arising out of state-based violence (FAT) based on the Uppsala Conflict Data Program (Gleditsch et al. 2002; Pettersson and Öberg 2020) appropriate.

Regarding the measurement of a political or security threat, I performed a sensitivity test of the empirical results by replacing the condition CAP with a combined score of the target’s geographical distance in kilometers from Brussels and the military strength of a country measured by the data for military expenditure in constant price (2017) in millions US Dollar (SIPRI 2020). The condition capabilities (CAP) was measured based on the military personnel of a country in the year of a sanctions decision or the year with the latest information available (2012 in the dataset). In a first step of the QCA analysis, I test for necessary conditions. As with the condition CAP, no condition passes the threshold of 1.000 in order to be considered a necessary condition for an economic design of EU sanctionsThe analysis of sufficiency delivers a slightly more complex conservative solution term when replacing CAP with THREAT, but the results are similar from an empirical perspective. According to the conservative solution term, severe human rights violations constitute part of three pathways and can therefore be considered a part of the sufficient conditions triggering the adoption of EU economic sanctions. Similarly, THREAT features in two pathways as part of the sufficient constellation of conditions in combination with either presence of US sanctions (USE) or the saliency of a conflict (SAL). This resonates with the findings when we employ CAP rather than THREAT. The first pathway covers Russia and Ukraine3 (Crimea and Sevastopol) uniquely, the second pathway covers Myanmar/Burma2 uniquely, and both cover the case of Iran. By contrast to the solution term produced with CAP rather than THREAT, here we observe a third pathway covering uniquely the case of Syria through a combination of grave human rights violations, the presence of US sanctions, and high saliency of the conflict. The consistency, PRI, and coverage scores are at a maximum of 1.000. As with CAP as a condition, Venezuela is covered by a fourth pathway of the conservative solution term. The intermediate and parsimonious solution terms are robust and identical to the solution terms as reported in the research article when replacing CAP with THREAT at maximum consistency, PRI, and coverage scores.

*Alternative conditions: sanctions’ duration*

The duration of sanctions’ episodes is a likely condition to impact the design of EU restrictive measures. Since sanctions can be considered a tool of coercive diplomacy whereby actors increasingly pressure targets into changing a certain behavior we might expect an intensified sanctions’ escalation in cases of a target’s non-compliance. With an expanding duration of sanctions in force against a particular target we would therefore expect an increasing escalation of the measure’s design. Indeed, sanctions’ duration and its impact has received attention from a wide array of scholarly research (e.g. Dizaji and Bergeijk 2013; Jeong 2019).

An underlying assumption of sanctions’ duration and an expansion of the sanction’s respective design is that actors gradually escalate a certain measure by increasing the costs imposed on the target. Economic sanctions usually imply larger costs for the target than targeted sanctions (see section on ‘Conceptualizing the design of sanctions’ in the research article). Based on this assumption, I expect an economic design of an EU restrictive measure to be accompanied by a gradual adjustment of the respective sanction program. I call this condition gradualism (GRAD).

In line with previous research (Portela and van Soest 2012), I rely on the temporal sequence of Council decisions regarding the imposition of a sanction regarding the calibration of gradualism (GRAD). When the EU, acting through the Council, adopted restrictive measures decisions over a sequence of multiple, at least two, years, I coded GRAD a 1. When the EU, through the Council, adopted restrictive measures on a particular target in one year only, GRAD was coded a 0. In this way, the calibration addresses the temporal dimension of sanctions and at the same time it is adequate for a crisp-set QCA analysis.

Here, I report the results of the QCA analysis when including gradualism (GRAD) as a condition. In a first step of the set-theoretic analysis, I test for necessary conditions. When including GRAD, none of the conditions passes the threshold of 1.000 in order to be considered a necessary condition. In a second step of the set-theoretic analysis, I test for sufficient conditions. When doing so, the conservative solution term displays the three pathways as identified in the research article with the addition of gradualism in the first two pathways. This implies that the empirical findings of the research article remain robust with the addition of GRAD as part of the combination of sufficient conditions.

When exploring the gradualism of EU restrictive measures in the set under investigation, however, I hesitate to interpret this finding empirically. Of the country cases under investigation, five are in the set GRAD, i.e. in five sanctions cases the EU imposed restrictive measures gradually over a minimum of two years. These cases are Belarus, Iran, Myanmar/Burma2, Russia, and Syria. Examining those cases, the concrete dynamics of how the gradual adjustment of sanctions unfolded vary to a large extent. Firstly, in the cases of Belarus and Myanmar/Burma2, the temporal distance is large. A first set of decisions by the EU was taken in 2006 and 2013 respectively and followed by a second set of decisions in 2011 and 2018 respectively. In the three other cases, Iran, Russia, and Syria, the temporal distance is only one year. Secondly, in some sanctions decisions like the restrictive measures imposed on Russia or Syria, the EU started with an economic design straight away and increasingly expanded the measures over time. In other sanctions decisions like Belarus, the EU stuck to targeted measures which it gradually expanded. Given the variation of how gradualism (GRAD) plays out in the concrete empirics of EU design decisions on sanctions, I hesitate to interpret it as a sufficient condition for an economic sanction’s design.

*Absence of economic sanctions*

According to the QCA protocol and in order to maximize robustness, the QCA analysis of the outcome, i.e. economic sanctions, is followed by an analysis of the outcome’s absence, i.e. targeted sanctions in the context of this research article. Here, I report the respective empirical results of the QCA analysis regarding the absence of economic sanctions (~ECON). Regarding the measurement of ~ECON as well as the calibration of the conditions military capabilities (CAP), fatalities (FAT), US sanctions (USE), and a conflict’s saliency (SAL), I refer the reader to the methods section in the research article.

A first step in the set-theoretic analysis is the test for necessary conditions. None of the conditions passes the consistency threshold of 1.000, but the absence of US economic sanctions (~USE) and of a conflict’s saliency (~SAL) fulfill the 1.000 consistency threshold each. The RoN and coverage scores are at 0.833 and 0.500 in the case of RoN for USE and SAL respectively and 0.929 and 0.812 in the case of coverage for USE and SAL respectively.

A second step in the set-theoretic analysis is the creation and minimization of the truth table in order to investigate sufficient conditions. At maximum consistency, PRI, and coverage scores of 1.000 the conservative solution term identifies one pathway towards EU non-economic sanctions. This pathway combines the absence of US economic sanctions and the absence of a conflict’s saliency (~SAL):

~USE*~SAL > ~ECON

The combination of absent US economic sanctions and a low or non-existing saliency of the conflict is, thus, necessary and sufficient for the EU adopt targeted rather than economic sanctions. At a maximum coverage score, this solution term covers the entire range of ~ECON cases, i.e. Belarus, Bosnia and Herzegovina, Burundi, Egypt, Guinea, Moldova, Myanmar/Burma1, Nicaragua, Tunisia, Ukraine, Ukraine2, Zimbabwe 1, Zimbabwe2. Interpreting this finding empirically, the EU turns to a targeted rather than an economic design of sanctions when the US does not pioneer with economic measures and when the conflict has low saliency only.

Figure 2: Comparison of EU (1989-2019), US (1950-2015), and UN (1991-2013) sanctions

Reference: own compilation based on Biersteker et al. (2018), Giumelli et al. (2020), and Weber and Schneider (2020)

Figure 3: Designs of EU sanctions (1989-2018)

Reference: own illustration based on Giumelli et al. (2020)

**References**

Biersteker, T. J., Eckert, S. E., Tourinho, M., & Hudáková, Z. (2018). UN Targeted Sanctions Datasets (1991-2013). *Journal of Peace Research, 55*(3), 404-12.

Binder, M. (2015). Paths to intervention: What explains the UN’s selective response to humanitarian crises? *Journal of Peace Research, 52*(6), 712-726.

Dizaji, S. F., & Bergeijk, P. A. G. (2013). Potential early phase success and ultimate failure of economic sanctions: A VAR approach with an application to Iran. *Journal of Peace Research, 50*(6), 721-736.

Giumelli, F., Hoffmann, F., & Książczaková, A. (2020). The when, what, where and why of European sanctions. *European Security* doi: 10.1080/09662839.2020.1797685.

Gleditsch, N. P., Wallensteen, P., Eriksson, M., Sollenberg, M., & Strand, H. (2002). Armed Conflict 1946-2001: A New Dataset. *Journal of Peace Research, 39*(5), 615-637.

Jeong, J. M. (2019). Do sanction types affect the duration of economic sanctions? The case of foreign aid. *International Political Science Review, 40*(2), 231-245.

Kreutz, J. (2017). Human Rights, Geostrategy, and EU Foreign Policy, 1989-2008. *International Organization, 69*(1), 195-217.

Pettersson, T., & Öberg M. (2020). Organized violence, 1989-2019. *Journal of Peace Research, 57*(4).

Weber, P., & Schneider, G. (2020). Post-Cold War sanctioning by the EU, the UN, and the US: Introducing the EUSANCT Dataset. *Conflict Management and Peace Science*, doi: 10.1177/0738894220948729.
